# Normative data and associated factors of hand grip strength among elderly individuals: The Yilan Study, Taiwan

**DOI:** 10.1038/s41598-020-63713-1

**Published:** 2020-04-20

**Authors:** Po-Jung Pan, Ching-Heng Lin, Nan-Ping Yang, Hsi-Chung Chen, Hsuan-Ming Tsao, Pesus Chou, Nai-Wei Hsu

**Affiliations:** 1 Community Medicine Research Center & Institute of Public Health, School of Medicine, National Yang-Ming University, Taipei, Taiwan; 2 Department of Physical Medicine and Rehabilitation, National Yang-Ming University Hospital, Yilan, Taiwan; 3 Department of Medical Research, Taichung Veterans General Hospital, Taichung, Taiwan; 4 Department of Orthopedic Surgery, Hualien Hospital, Ministry of Health & Welfare, Hualien, Taiwan; 5 Department of Psychiatry & Center of Sleep Disorders, National Taiwan University Hospital, Taipei, Taiwan; 6 Division of Cardiology, Department of Internal Medicine, National Yang-Ming University Hospital, Yilan, Taiwan; 7 Public Health Bureau, Yilan County, Taiwan

**Keywords:** Health care, Medical research

## Abstract

Weak grip strength is associated with subsequent mortality in elderly populations. The normative data and associated factors of HGS in community-dwelling elderly Taiwanese populations require further evaluation. From February 2012 until the end of 2016, all residents of Yilan City, Taiwan aged 65 years or older were randomly selected for a population-based community health survey. A total of 2,470 older adults were enrolled in this study. The relationships between HGS and various anthropometric and sociodemographic correlates were examined. The results showed that HGS was higher in men than in women. The mean HGS exhibited a decreasing trend with advanced age in both men and women. HGS was significantly associated with height, weight, and exercise habits. The physical as well as the mental component summary measures of health-related quality of life (HRQoL) were positively associated with HGS. After HRQoL was integrated into the regression model, female sex, age, waist circumference, and diabetes mellitus were significantly negatively associated with HGS. In conclusion, HGS significantly decreased with advanced age. among community-dwelling Taiwanese elderly people, Various factors had different effects on HGS.

## Introduction

Hand grip and quadriceps muscle strength decrease with advanced age^1^. A cross-sectional analysis of 10,149 adults aged 60 years and older revealed that the prevalence of physical limitations, such as decrease of the activity of daily life (ADL) and the instrumental activity of daily life (IADL), was significantly higher among participants with low hand grip strength (HGS) than among those with high HGS^2^. Another recent systematic review of 21,197 participants demonstrated a relationship between low HGS and high hip fracture incidence in elderly people^3^. Moreover, a pooled analysis of 6,426 nondependent community-dwelling people aged 60 years and older revealed a 40% increase of the adjusted risk of all-cause mortality among elderly individuals with HGS in the lowest quartile; thus, weak grip strength was associated with subsequent mortality^4^. Therefore, HGS is a crucial indicator of health status and prognosis among elderly populations.

Normative data of grip strength across the life course were collected by Dodds *et al*. in Great Britain. After comparing these normative data with those of other world regions, they found that the grip strength measure obtained from people living in developing regions was clearly lower than that of people living in developed regions^5^. However, studies on HGS in Asia are few in number and were conducted later than those in Western countries. A 4-year longitudinal study recruited 3,018 community-dwelling Chinese individuals aged 64 years and older and found a more rapid decline in grip strength in women than in men. The study results also revealed that the grip strength in elderly people of Chinese descent was weaker than that in elderly people of African descent and those of European descent white people^6^. HGS among elderly people in Singapore was also lower than that among elderly people in some Western countries^7^. Kim *et al*. demonstrated that among Koreans HGS peaked at 35–39 years of age and thereafter decreased in both sexes^8^. Additional studies are warranted, especially in other regions of Asia, to evaluate the normative data of HGS.

Age, sex, race/ethnicity, education, smoking status, body mass index, comorbidities, and physical activity have been associated with HGS in community-dwelling elderly people^2^. Greater height, higher weight, and smaller waist circumference have also been independently associated with higher HGS^7^. Because HGS and its associated factors have become a major public health concern, especially among elderly populations worldwide, additional studies are required to thoroughly elucidate the various factors that influence HGS and the underlying associations. Therefore, the present study was designed to investigate the following: (1) the normative data of HGS and (2) the associated factors of HGS among community-dwelling elderly individuals in Taiwan.

## Methods

### Study design and participants

This study was part of the Yilan study series^9–13^, a population-based community health survey conducted by the Community Medicine Research Center of the National Yang-Ming University and the National Yang-Ming University Hospital in Taiwan. The study protocol was evaluated and approved by the Institutional Review Board (IRB) of National Yang-Ming University Hospital (IRB Approval No.: 2011A016). All the enrolled participants provided written informed consent. All methods were performed in accordance with the relevant guidelines and regulations.

The study methods have been described elsewhere^9,11,14^. In brief, from February 2012 to the end of 2016, all residents of Yilan City, located in the northeast of Taiwan, aged 65 years or older were randomly selected and invited to participate. Those who agreed to participate in the study were enrolled, and those who did not agree to participate were excluded. Additionally, those with preexisting hand problems were excluded from HGS measurement. Well-trained project assistants visited all the enrolled participants at their homes and conducted face-to-face interviews. Basic data and health-related information, including demographic characteristics, body weight/height, waist circumference, education level, living status (full-time living alone, part-time living alone, and not living alone), lifestyle (ie, smoking/drinking habits, exercise condition within the past 1 week, and sleep status), health-related quality of life (HRQoL) within the past 1 month, self-reported medical histories of chronic diseases (diabetes mellitus, hypertension, cardiovascular disease, hyperlipidemia, cerebrovascular accident, gout, and cataract) and current psychological status, and fall episodes within the past 1 year, were collected through physical examination and well-established questionnaires^9,11,14^. The Short-Form 12 Health Survey Version 2 (SF-12v2) was used for evaluating the HRQoL; the SF-12v2 comprises 12 items that constitute the mental component summary (MCS) and physical component summary (PCS) for measuring the participant’s mental and physical function status in the past 4 weeks^15,16^.

Initially, 2,584 community-dwelling elderly individuals were randomly selected and interviewed. However, 114 of them were determined to have mild to moderate disability because of a previous cerebrovascular accident. Therefore, these participants were excluded from the present study. Finally, 2,470 subjects were enrolled.

### Measurement of HGS

HGS of both hands was measured with a hydraulic hand dynamometer (Jamar, Jackson, MI, USA). Each participant completed the trial for each hand, and the final estimate of HGS was the average of all measurements^10,17^.

### Statistical analysis

Descriptive statistics are presented as numbers of cases, percentages, and means with standard deviation (SD). The independent *t* test and the chi-squared test were used to analyze the differences in continuous and categorical variables, respectively, between groups. Box plots of HGS estimates were drawn, and their trend tests were evaluated using a simple linear regression. The beta (β) coefficient and the partial R-squared of individual independent factors of HGS were calculated using stepwise multivariate linear regression to perform multivariate analyses.

## Results

### Sociodemographic data of participants

A total of 2,470 adults aged 65 years and older [998 (40.4%) men and 1,472 (59.6%) women] were enrolled in this study. A comparison of the basic characteristics, including age strata, chronic physical disorders, living status, and education level, of the sexes is presented in Table 1. The sex distribution, especially in the 65–69 years stratum, was significantly different. However, no significant difference in comorbidities such as heart disease (32.5% vs 31.9%) and diabetes mellitus (22.3% vs 24.7%) was noted between men and women. Additionally, men exhibited lower rates of full-time and part-time living alone and higher rates of not living alone than women (5.4% vs 9%; 2.4% vs 5.4%; 92.2% vs 85.6%, respectively). Furthermore, men also had higher education levels (junior high school or higher education level; 44.9% vs 23.0%).

**Table 1:** Basic characteristics of the enrolled elderly participants.

	Total (n = 2470)		Male		Female		p value of χ^2^ test^a^
	No.	%	No.	%	No.	%	
Gender							
male	998	40.4	-----	-----	-----	-----	
female	1472	59.6	-----	-----	-----	-----	
Age Stratum (years)							
65–69	270	10.9	61	6.1	209	14.2	<0.001
70–74	711	28.8	269	27.0	442	30.0	
75–79	686	27.8	292	29.3	394	26.8	
80 or more	803	32.5	376	37.7	427	29.0	
Chronic Physical Disorders							
Heart disease (yes)	793	32.1	324	32.5	469	31.9	0.761
DM (yes)	586	23.7	223	22.3	363	24.7	0.184
Living Status							
full-time living alone	186	7.5	54	5.4	132	9.0	<0.001
part-rime living alone	104	4.2	24	2.4	80	5.4	
not living alone	2179	88.3	920	92.2	1259	85.6	
Educational Level							
junior high school or higher	785	31.8	447	44.9	338	23.0	<0.001
none or primary school	1681	68.2	548	55.1	1133	77.0	

Abbreviations: DM: diabetes mellitus.

Notes: ^a^Comparison between sexes.

### Normative Data of HGS

Table 2 presents the means and SD of HGS by sex and age stratum. The mean values of HGS for men were significantly higher than those for women in sum (25.4 kg vs 15.5 kg) or in subgroups by 2- and 4-year age strata. The mean HGS dropped from 33.0 kg in men aged 65–69 years to 21.1 kg in men aged 80 years or older. Moreover, HGS dropped from 19.1 to 12.6 kg in women. In the oldest stratum (75 years or older), the mean HGS values decreased to only 23.2 and 13.9 kg in men and women, respectively.

**Table 2:** Means and standard deviations (SD) of hand grip strength (HGS; kg) by sex and age stratum.

	Total			Male			Female			p value of t test^a^
	No.	mean	SD	No.	mean	SD	No.	mean	SD	
**In General** (aged 65 years or more)	2470	19.5	8.4	998	25.4	8.6	1472	15.5	5.4	<0.001
Age Stratum (years)										
* 4 strata*										
65–69	270	22.3	7.9	61	33.0	6.8	209	19.1	4.8	<0.001
70–74	711	21.5	8.6	269	29.0	7.9	442	16.9	5.1	<0.001
75–79	686	19.8	8.2	292	25.9	7.8	394	15.3	4.7	<0.001
80 or more	803	16.6	7.8	376	21.1	7.8	427	12.6	5.1	<0.001
* 2 strata*										
65–74	981	21.7	8.4	330	29.8	7.8	651	17.6	5.1	<0.001
75 or more	1489	18.1	8.1	668	23.2	8.2	821	13.9	5.1	<0.001

Notes: ^a^Comparison between sexes.

**Figure 1:**
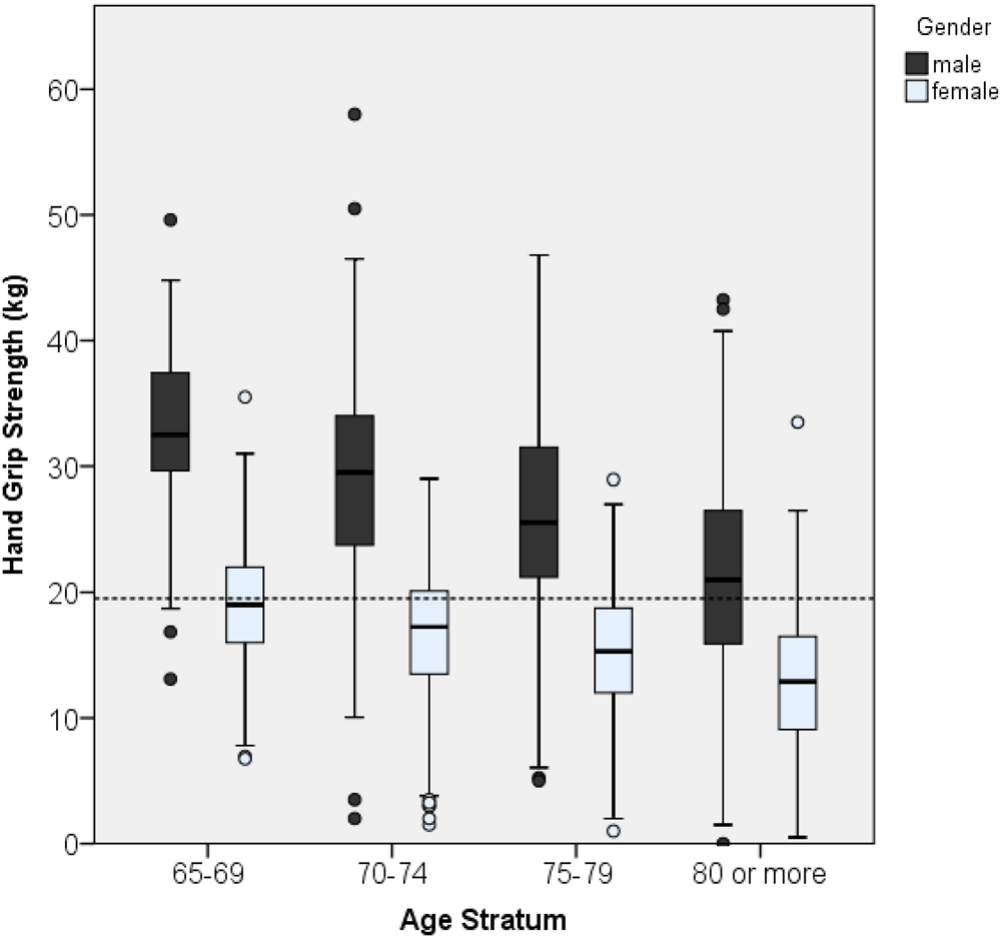
Box plots of HGS estimates in four age strata subgroups among men and women; trend test showing a p value of <0.001 for men and <0.001 for women.

**Figure 2:**
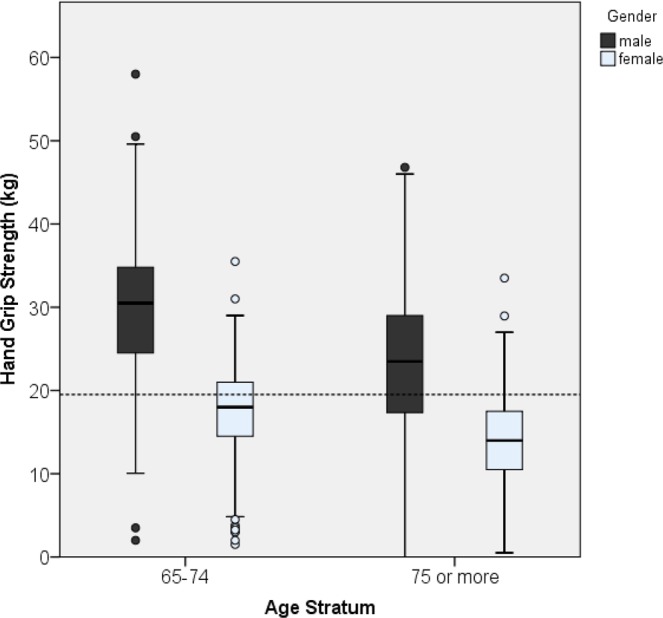
Box plots of HGS estimates in two age strata subgroups among men and women; trend test showing a p value of <0.001 for men and <0.001 for women.

Minimum, maximum, median, and interquartile range of all HGS point estimates among various age strata for both sexes are presented in Figs. 1 and 2. Significantly decreasing trends (p < 0.001) of HGS across all age strata were noted in both men and women. The medium values of HGS in men in all age strata were higher than those in women.

### Correlates of HGS

Stepwise multivariate linear regression analysis was conducted to evaluate the correlations of HGS with various factors. The results are presented in Table 3. For the overall sample, HGS was significantly positively associated with height (β = 0.19) and weight (β = 0.18) and slightly associated with exercise. Female sex, age, waist circumference, diabetes mellitus, and heart disease were independently associated with decreased HGS (β = −7.5, −0.35, −0.12, −1.16, and −0.63, respectively). HGS was significantly higher in those living alone full time (β = 1.17). Stratified regression analysis in men and women indicated that HGS was positively associated with height (β = 0.24 and 0.16) and weight (β = 0.30 and 0.13) and negatively associated with age (β = −0.53 and −0.23), waist circumstance (β = −0.24 and −0.09) and diabetes mellitus (β = −1.33 and −0.10) in both genders, and heart disease in women (β = −0.64). The HGS was significantly stronger (β = 2.72) in men with full-time living alone status. For the overall sample, the highest R-squared percentage (36.4% in a total of 53.3%) was noted for height. In men and women, the highest R-squared percentage values were obtained for age (20.9% in a total of 35.8% and 16.1% in a total of 26.1%).

**Table 3:** Correlations of HGS analyzed using stepwise multivariate linear regression.

	Total^a^			Male^b^			Female^b^		
	Beta (β) coefficient	Partial R-square (%)	P value	Beta (β) coefficient	Partial R-square (%)	P value	Beta (β) coefficient	Partial R-square (%)	P value
Height (cm)	0.19	36.4%	<0.001	0.26	9.8%	<0.001	0.15	5.7%	<0.001
Gender (male as reference)	−7.50	5.6%	<0.001						
Age (years)	−0.35	8.7%	<0.001	−0.53	20.9%	<0.001	−0.23	16.1%	<0.001
Exercise (mins), in 1 week	0.00	0.7%	<0.001	0.00	0.8%	0.002	0.00	1.4%	<0.001
Weight (kgw)	0.18	0.4%	<0.001	0.30	0.7%	<0.001	0.13	0.6%	<0.001
Waist circumference (cm)	−0.12	0.9%	<0.001	−0.24	2.6%	<0.001	−0.09	1.3%	<0.001
DM (yes vs no)	−1.16	0.4%	<0.001	−1.33	0.5%	0.014	−1.00	0.7%	0.001
Heart disease (yes vs no)	−0.63	0.1%	0.015				−0.64	0.3%	0.016
Living Status									
full-time living alone	1.17	0.2%	0.008	2.72	0.6%	0.005			
part-time living alone	0.46		0.430	−0.74		0.606			
not living alone				Reference					
Model R-square	53.3%			35.8%			26.1%		

Abbreviation: DM: diabetes mellitus

Notes: ^**a**^The model was adjusted using regression with stepwise selection for sex, age, heart disease, DM, living status, education level, height, weight, waist circumference, and exercise.

^b^The model was adjusted using regression with stepwise selection for age, heart disease, DM, living status, education level, height, weight, waist circumference, and exercise.

A stepwise multivariate linear regression analysis was performed again to evaluate the associated factors of HGS with the inclusion of HRQoL measured using the SF-12v2 with MCS and PCS. For the overall sample, HGS was significantly associated with height (β = 0.19), PCS (β = 0.19), MCS (β = 0.16), and weight (β = 0.15) and slightly associated with exercise after adjustment. Similarly, female sex, age, waist circumference, and diabetes mellitus were independently associated with decreased HGS (β = −7.18, −0.31, −0.10, and −0.93, respectively). Stratified regression analysis in men and women demonstrated that HGS was positively associated with height (β = 0.24 and 0.16), PCS (β = 0.24 and 0.14), MCS (β = 0.21 and 0.12), and weight (β = 0.27 and 0.11) and negatively associated with age (β = −0.47 and −0.21), waist circumstance (β = −0.20 and −0.07), and diabetes mellitus (β = −1.04 and −0.08). After controlling for height, age, PCS, MCS, weight, waist circumstance, and diabetes mellitus, men with full-time living alone status still had significantly stronger HGS (β = 2.35). For the overall sample, height had the highest R-squared percentage (36.4% in a total of 57.8%). In men and women, the highest R-squared percentage values were related to age (20.6% in a total of 42.8% and 16.1% in a total of 33.1%).

## Discussion

HGS is a crucial and easily available indicator for predicting the ability to perform daily functions, especially among elderly individuals^18^. In our study, 2,470 community-dwelling elderly persons were recruited and we conducted physical examination and face-to-face interviews at their homes^11,14,17^. Unlike the other studies in Taiwan, we used primary data collection with a large sample size^5,19–21^. Therefore, elderly individuals who previously tended to stay at home because of disability and could not go outside to undergo examinations could be assessed. Thus, even in the oldest age stratum (80 years or older), 803 residents were recruited in this study.

Comparing the recently published normative data, we determined that the HGS of people aged 65 years or older in our study was lower than that evident in studies from European regions. For example, the mean values were approximately 13% lower than the results of the age-matched groups presented by Dodds *et al*. in 2014^22^. We also compared our results with the data obtained from other studies in Taiwan and determined that our mean values were similar to those reported by Wu *et al*. in 2009^20^, but slightly lower than the data reported by Liao *et al*. in 2014^23^. A comparison of our data with those reported in other Asian regions revealed that the mean values obtained in our study were lower than those obtained in the studies by Seino *et al*. in Japan and Kim *et al*. in Korea^8,24^, but were similar to those of another study reported by Ong *et al*. in Singapore in 2017^7^. It has been proposed that global variations in the norms of HGS were present, which might be attributable to the status of development^5^. Additionally, HGS has been determined to decrease with advanced age and among women^1,4,5,22,25^. After controlling for age and sex, several other factors have also been proposed to influence HGS, such as anthropometric elements (body weight, height, limb circumference, and palm length), testing protocol, study methods, measuring device, comorbidities, and other socioeconomic factors (ethnicity, education, marital status, and employment status)^5,7,19,23,24^. In our study, we proposed that the anthropometric differences might play a crucial role in the lower norms of HGS compared with the values reported for Caucasians in other studies because people in Taiwan have smaller physical stature. Additionally, it was reasonable to estimate that elderly individuals who accepted HGS measurement at certain community centers, similar to those in Liao’s study, might have a more favorable health status because of their independent ambulation. By contrast, those who received interviews at home in our study might include those individuals with poor mobility and worse health status and with resultant lower HGS. This would indicate that the real norm of HGS in Taiwan might be lower than those reported in other studies.

HGS is a diagnostic criterion for sarcopenia, and the cutoff value in Asian populations has been proposed to be <26 kg for men and <18 kg for women. In our study, the cutoff points for both sexes were higher than the mean values (25.4 kg for men and 15.5 kg for women)^26^. As evident in Table 2, it is clear that men older than 75 years and women older than 70 years had mean HGS values below the threshold of sarcopenia. A higher percentage of elderly people in our study population might explain the lower HGS values obtained in our study.

In Figs. 1 and 2 illustrate the large gap between maximum and minimum HGS in each age stratum. Furthermore, the coefficient of variation increased with advanced age. We proposed that the increased variation in HGS with advanced age might be attributable to the accumulation of those correlation factors. Lino contended that various chronic diseases might be associated with low HGS, and thus in many elderly people, an increase in the number of comorbidities might lead to the large variation of HGS^25^.

In our study, age, sex, height, weight, hypertension, and diabetes were significantly associated with HGS, which was compatible with the results reported by Lino and Ramlagan^25,27^. As presented in Table 3, for each year increase of age after 65 years, an average reduction of 0.35 kg in HGS can be expected. A decrease of 3.5 kg in HGS for each decade of life can be estimated, which is substantially higher than that observed by Lino in Brazil^25^. Particularly, men exhibited a larger decline in HGS than women for each year of increase (0.53 kg vs 0.23 kg). Therefore, prevention of muscle strength deterioration in elderly people, especially in men, should be a focus of interventions after retirement from their careers.

Although weight was positively associated with HGS, a negative association between waist circumference and HGS was also observed. Our result was consistent with the finding reported by Ong *et al*. in 2017^7^. This result suggested that older adults with a higher weight might have higher HGS; however, this did not hold true for obese older adults. Elderly people with a higher body weight consisting of lean muscle rather than central fat had higher HGS.

Exercise may improve muscle strength in elderly people^28^. As presented in Table 3, exercise in the past 1 week was a significant factor associated with HGS but with a low beta coefficient. According to our records, most elderly individuals performed low- or low-to-moderate-intensity physical exercise such as walking, which was apparently not sufficient to improve HGS. Therefore, health-related specialists should design appropriate beneficial programs for promoting physical fitness improvement among elderly individuals.

Furthermore, also evident in Table 3, heart disease and full-time living alone status were significantly associated with HGS. However, after the introduction of HRQoL (Table 4), the association of HGS with both the factors disappeared and the total R-squared increased. HRQoL is an objective measure of the consequence of health status on quality of life. It measures the effects of various chronic illnesses, treatments, and disabilities on the physical and mental status experienced in daily life^29^. Therefore, the PCS of HRQoL had a positive association with HGS, and HRQoL might mediate the effects of heart disease and living status on HGS^24^. However, mental status has often been overlooked as one of the associated factors of HGS in elderly people. Depression has ever been observed to be negatively associated with HGS^25,30^. Pasco suggested that sarcopenia with resultant decreased strength and depression possibly shared a common pathophysiological pathway^31^. We have also previously demonstrated that depression is negatively associated with both MCS and PCS in elderly people^11,14^. Therefore, it would not be surprising for MCS to be positively associated with HGS.

**Table 4:** Correlations of HGS analyzed using stepwise multivariate linear regression and including HRQoL measures.

	Total^c^			Male^d^			Female^d^		
	Beta (β) coefficient	Partial R-square (%)	P value	Beta (β) coefficient	Partial R-square (%)	P value	Beta (β) coefficient	Partial R-square (%)	P value
Height (cm)	0.19	36.4%	<0.001	0.24	9.8%	<0.001	0.16	5.7%	<0.001
Gender (male as reference)	−7.18	5.6%	<0.001						
Age (years)	−0.31	8.7%	<0.001	−0.47	20.9%	<0.001	−0.21	16.1%	<0.001
PCS score^**a**^	0.19	3.5%	<0.001	0.24	5.4%	<0.001	0.14	5.4%	<0.001
MCS score^**b**^	0.16	2.2%	<0.001	0.21	3.4%	<0.001	0.12	3.9%	<0.001
Weight (kgw)	0.15	0.4%	<0.001	0.27	0.7%	<0.001	0.11	0.4%	<0.001
Waist circumference (cm)	−0.10	0.6%	<0.001	−0.20	1.9%	<0.001	−0.07	0.9%	<0.001
DM (yes vs no)	−0.93	0.2%	0.001	−1.04	0.3%	0.044	−0.80	0.4%	0.004
Exercise (mins), in 1 week	0.00	0.1%	0.010				0.00	0.3%	0.008
Living Status									
full-time living alone				2.35	0.5%	0.010			
part-time living alone				−0.94		0.484			
not living alone				Reference					
Model R-square	57.8%			42.8%			33.1%		

Abbreviation: QoL: quality of life; DM: diabetes mellitus.

Notes: ^**a**^Physical component summary (PCS) measure of quality of life obtained using short-form (SF)−12 questionnaire.

^**b**^Mental component summary measure (MCS) of quality of life obtained using SF-12 questionnaire.

^**c**^The model was adjusted using regression with stepwise selection for sex, age, heart disease, DM, living status, education level, height, weight, waist circumference, exercise, PCS score, and MCS score.

^d^The model was adjusted using regression with stepwise selection for age, heart disease, DM, living status, education level, height, weight, waist circumference, exercise, PCS score, and MCS score.

Chronic diseases have been demonstrated to be associated with HGS^12,13,15,25^. In our study, both diabetes and heart disease were associated with HGS. Even with the introduction of HRQoL, diabetes exhibited a significantly negative association with HGS. Stenholm suggested that insulin resistance might have catabolic effects on muscle and resultant sarcopenia^13^. Mainous also suggested that sarcopenia and impaired metabolic states share common cellular and molecular characteristics^12^. These findings might partially explain the direct association between diabetes and HGS.

Our study analyzed the data from a population-based community health survey in Yilan City. For a more comprehensive survey, we have conducted a home-visit intervention. However, there were still some limitations in this study. First, some factors related to muscle strength, such as the nutritional status of the participants, were not included in this study. These factors may be included in future following-up studies with home-visit interventions^32^. Second, for the HGS test, most of the older adults took the measurements in a standing position, with the exception of some older people who took the procedure in a sitting position to prevent falls. Although little difference was evident between these two test positions^23^, a more suitable sitting position should be applied to reduce the small intervention error in future research. Third, though the same two well-trained assistants could minimize the investigation bias, it took longer time for them to recruit these data from over 2,000 elderly individuals. Therefore, some changes of the community at macro level, such as the health policy of the government and the socioeconomic status of the inhabitants, might happen and affect these individuals’ health outcomes during these 4 years. Forth, under the restriction of Personal Information Protection Act in Taiwan, we were not allowed to have the detailed names and addresses of these elderly individuals who were registered in Yilan city. Therefore, we could not but randomly selected and invited the old inhabitants to participate this study. It was not surprising that there were some differences when comparing the distribution of age and sex of these participants to that of the registered inhabitants in Yilan city^33^. This difference may limit the generalizability of our findings to the whole population in Taiwan. However, on the other hand, the elderly who agreed to participate our study might be the population who were prone to receive further community interventions. Therefore, the results of this study will provide us more sound data to plan and perform the following health promotion programs in the near future. Finally, some older adults did not participate in interviews for personal reasons, especially those in the age stratum of 65–69 years. Including more participants, especially in this age stratum, is necessary to obtain reliable results in the following-up studies. Despite these limitations, the results from this study provide important and valuable information for the government to arrange further steps to increase the health status among elderly people in the community.

## Conclusion

Among community-dwelling Taiwanese elderly individuals, a significant decreasing trend of HGS with advanced age has been noted both in men and women. The HGS was independently positively associated with weight, height, PCS, MCS, and exercise habit and negatively associated with age, female sex, waist circumference, and a history of diabetes mellitus. Additionally, because this study recruited more elderly people with disabilities, the results suggested that the normative data of HGS in elderly Taiwanese people might be lower than those reported from previous studies.

## Data Availability

The datasets analyzed during the current study are available from the corresponding author on reasonable request.
